# Comparison of specialized stent versus generic stent and bare stent combination for transjugular intrahepatic portosystemic shunt creation

**DOI:** 10.1038/s41598-024-64358-0

**Published:** 2024-06-23

**Authors:** Yaowei Bai, Jiacheng Liu, Chaoyang Wang, Wei Yao, Shuguang Ju, Yingliang Wang, Chen Zhou, Xiangjun Dong, Chuansheng Zheng

**Affiliations:** 1 Department of Radiology, Union Hospital, Tongji Medical College, Huazhong University of Science and Technology, Jiefang Avenue #1277, Wuhan, 430022 China; 2 Hubei Province Key Laboratory of Molecular Imaging, Wuhan, 430022 China

**Keywords:** Transjugular intrahepatic portosystemic shunt, Stent, Shunt dysfunction, Liver cirrhosis, Portal hypertension

## Abstract

Transjugular intrahepatic portosystemic shunt (TIPS) creation using the Viatorr stent remains relatively uncommon in underdeveloped and high-burden disease regions in Asia–Pacific, and there is a lack of comparative studies regarding its prognostic effects compared with the generic stent-graft/bare stent combination. The purpose of this retrospective study is to compare the prognostic endpoints of these two treatments in patients who underwent TIPS creation. Clinical data from 145 patients were collected, including 82 in the combination group and 63 in the Viatorr group. Differences in prognostic endpoints (shunt dysfunction, death, overt hepatic encephalopathy [OHE], rebleeding) between the two groups were analyzed using Kaplan–Meier curves. The Cox proportional hazards model was used to identify independent risk factors for post-TIPS shunt dysfunction. The TIPS procedure was successful in all patients. After TIPS creation, both groups showed a significant decrease in porto-caval pressure gradient compared to that before TIPS creation. The stent patency rates at 6, 12, and 18 months were high in both the combination and Viatorr groups (93.7%, 88.5%, and 88.5% vs. 96.7%, 93.4%, and 93.4%, respectively). The stent patency rates was higher in the combination group than in the Viatorr group, although not statistically significant (HR = 2.105, 95% CI 0.640–6.922, Log-rank *P* = 0.259). There were no significant differences in other prognostic endpoints (death, OHE, rebleeding) between the two groups. The Cox model identified portal vein diameter (HR = 0.807, 95% CI 0.658–0.990, *P* = 0.040) and portal vein thrombosis (HR = 13.617, 95% CI 1.475–125.678, *P* = 0.021) as independent risk factors for post-TIPS shunt dysfunction. The shunt patency rates between the Viatorr stent and the generic stent-graft/bare stent combination showed no significant difference and the generic stent-graft/bare stent combination may be a viable alternative in areas where the Viatorr stent is not yet available.

## Introduction

Portal hypertension in liver cirrhosis is a common chronic liver disease worldwide that can lead to esophagogastric varices, hepatic encephalopathy, hepatorenal syndrome, and refractory ascites^1^. Transjugular intrahepatic portosystemic shunt (TIPS) plays an increasingly important role in reducing portal vein pressure and treating these complications by creating a shunt between the hepatic vein and portal vein^2^. The use of bare stents in TIPS was initially limited by shunt dysfunction, but the development of polytetrafluoroethylene covered stents greatly improved long-term patency^3,4^. The Viatorr stent, a self-expanding covered stent consisting of a 4–8 cm hepatic covered segment and a 2 cm portal vein bare segment, had been shown in large clinical studies to have excellent long-term patency and was widely used in Europe and America^5,6^. However, the Viatorr stent was introduced relatively late into the underdeveloped and high-burden disease regions in Asia–Pacific, primarily due to factors such as pricing and policies. Before the introduction of the Viatorr stent in China, the most common TIPS stents used were the generic stent-graft/bare stent combination^7^. Compared with bare stents, Viatorr stents can reduce the probability of stent obstruction^8^. However, at present, there exists a lack of comparative studies regarding the effectiveness of the Viatorr stent versus the generic stent-graft/bare stent combination on patient outcomes. This information is crucial for clinical decision-making in areas where the Viatorr stent is not yet available. Therefore, the purpose of this study was to explore the impact of Viatorr stent and the generic stent-graft/bare stent combination on patient outcomes.

## Methods

### Patients

**Figure 1:**
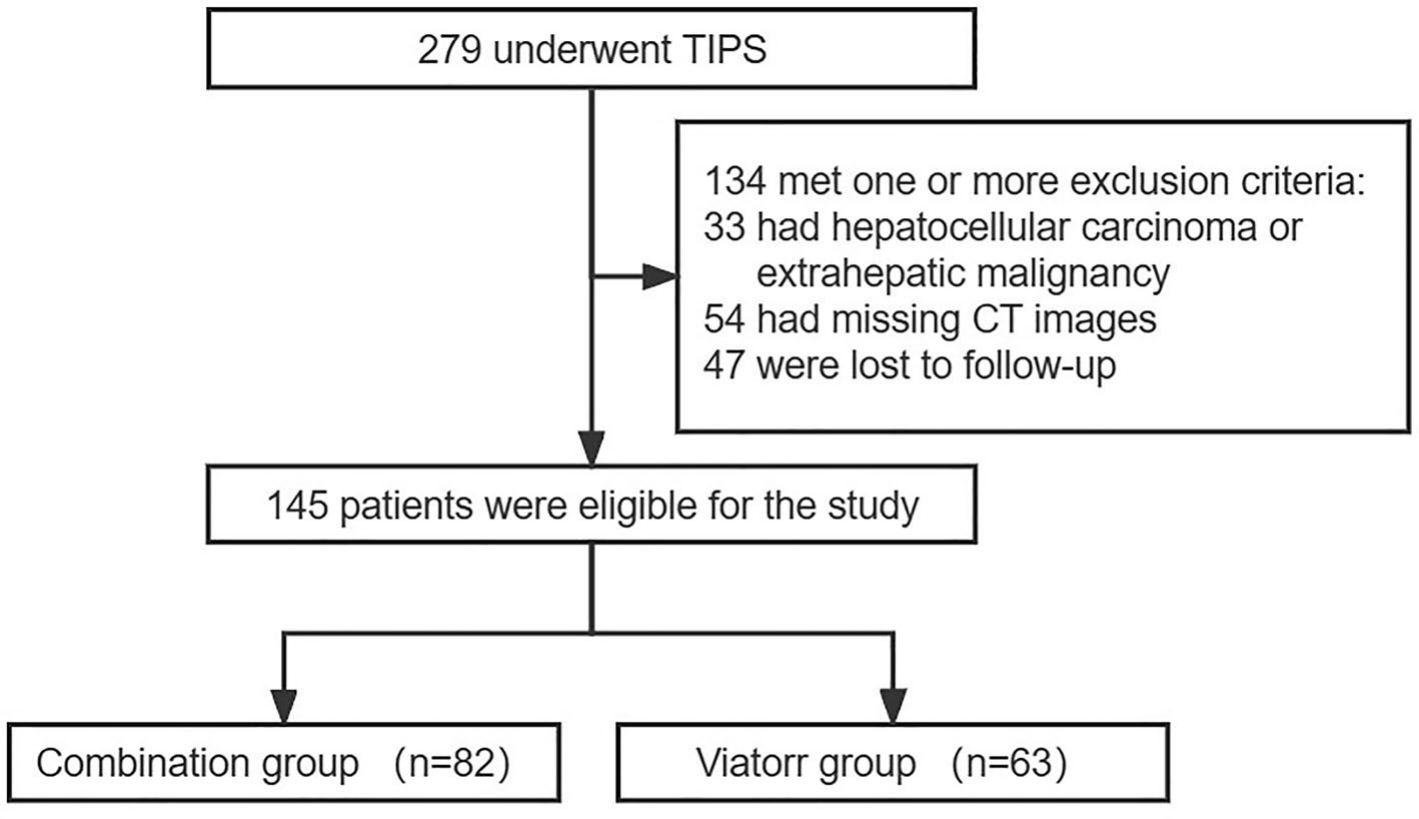
Flowchart of study participants.

We retrospectively collected clinical data of 279 patients who underwent TIPS creation at Union Hospital between February 2021 and February 2023. Eligible patients had confirmed portal hypertension due to liver cirrhosis. We excluded patients with incomplete computed tomography (CT) data, liver or extrahepatic tumors, and those without post-TIPS follow-up data (Fig. 1). This study was approved by the Institutional Review Board (IRB) of Union Hospital, Tongji Medical College, Huazhong University of Science and Technology and was conducted according to the tenets of the 1975 Declaration of Helsinki. Written informed consent was revoked for this retrospective study by IRB of Union Hospital, Tongji Medical College, Huazhong University of Science and Technology.

### Data collection and follow-up protocol

We collected clinical data for each patient, including gender, age, underlying cause of liver cirrhosis, reasons for TIPS creation, imaging data (such as portal vein diameter, portal vein thrombosis (PVT), spontaneous portosystemic shunts, and pre-and post-TIPS porto-caval pressure gradient (PPG)), biochemical examination (such as total bilirubin, albumin, alanine aminotransferase, aspartate aminotransferase, Creatinine, blood urea nitrogen, sodium, prothrombin time, international normalized ratio, platelet count), and any other relevant clinical data. We conducted clinical follow-up at the time points of 3, 6, 12, and 18 months after TIPS creation, including clinical evaluation, biochemical examination, Doppler ultrasound, and/or enhanced CT scan, depending on the patient’s condition.

### Clinical outcomes

The study involved the following endpoints such as rebleeding, overt hepatic encephalopathy (OHE), shunt dysfunction, and death. Shunt dysfunction was defined as imaging-confirmed stent lumen stenosis of 50% or greater^4^. This study did not consider a post-TIPS PPG higher than 12 mmHg as the criterion for shunt dysfunction. Patients who had PPG reduction greater than 20% but final PPG higher than 12 mmHg should not be categorized as having shunt dysfunction. Rebleeding was defined according to the Baveno VII consensus criteria^9^. Hepatic encephalopathy was classified into five grades according to the West Haven classification, and only levels II-IV were considered^10^.

### TIPS procedure

**Figure 2:**
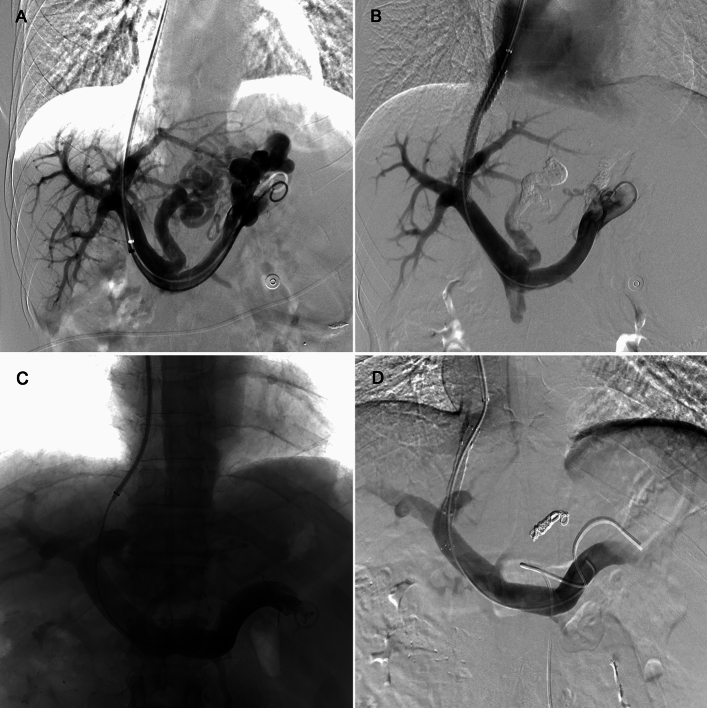
Representative images of TIPS procedure. (**A** and **B**), representative images from a patient in the combination group. Portal venography was performed after successful puncture and stent placement. (**C** and **D**), representative images from a patient in the Viatorr group. Portal venography was performed after successful puncture and stent placement.

The TIPS creation method was as described in previous studies^11^ (Fig. 2). The Cook RUPS-100 puncture device was used to puncture the right internal jugular vein, and a catheter was inserted through a sheath in the right internal jugular vein to reach the hepatic vein. Under fluoroscopy, the portal vein was punctured, and a direct channel between the hepatic vein and portal vein was established. A balloon (6-8mm) was used for dilation, and a stent was deployed to establish a shunt between the hepatic vein and portal vein. The type of stents used included the Viatorr stent-graft (Gore, USA) and the generic stent-graft/bare stent combination, which were selected by the interventionalist based on various factors, with the most important ones being inventory and price. When using the combination stents, a bare stent (Bard E-Luminexx vascular stent, Karlsruhe, Germany) was first placed in the intrahepatic tract, followed by the placement of a covered stent (Fluency; Bard Inc., USA, or Viabahn; Gore, USA) within the bare stent^11^. The PPG was measured before and after the shunt was established. The target of post-TIPS PPG was less than 12 mmHg for patients with variceal bleeding. Alternatively, a greater than 20% decrease from initial PPG could also be recommended as a target^2^. The choice of stent type and the decision to use auxiliary targeting methods, including percutaneous transhepatic portal vein target (PTPVT) and hepatic artery guide wire target (HAT) techniques, were at the discretion of the interventionalist based on the patient’s condition. Additionally, to reduce the occurrence of rebleeding, we also embolize large esophagogastric varices and splenorenal shunts simultaneously with TIPS placement.

### Statistical analysis

Statistical analysis was performed using SPSS (statistical product and service solutions, version 26.0, https://www.ibm.com/spss), GraphPad prism (version 10, https://www.graphpad.com/), and R (version 4.0.3, https://www.r-project.org/) software. Continuous variables were expressed as mean ± standard deviation (x ± s), and categorical variables were expressed as numbers and percentages (n [%]). Kaplan–Meier curves were plotted for comparing the differences in clinical endpoints. Cox regression analysis was used to evaluate the risk factors for shunt dysfunction, and variables with *P* < 0.2 in univariable analysis were included in multivariable analysis using the method of Forward LR. A nomogram was constructed based on the results of the Cox multivariable analysis to predict post-TIPS shunt dysfunction. A two-sided significance level of less than 0.05 was considered statistically significant.

### Ethics approval and consent to participate

This study was approved by the Institutional Review Board (IRB) of Union Hospital, Tongji Medical College, Huazhong University of Science and Technology and was conducted according to the tenets of the 1975 Declaration of Helsinki. Written informed consent was revoked for this retrospective study by IRB of Union Hospital, Tongji Medical College, Huazhong University of Science and Technology.

## Results

### Baseline characteristics of patients

In total, 145 patients were included in this study, with 82 and 63 patients in the combination and Viatorr groups, respectively (Table 1). The mean age of the patients was (58.1 ± 11.3) years, and 83 (57.2%) were male. Among all patients, 96 (66.2%) had hepatitis B virus, and 124 (85.5%) underwent TIPS creation due to variceal bleeding. The pre-TIPS albumin level was significantly lower in the combination group than in the Viatorr group (29.5 ± 5.3 g/L vs. 31.3 ± 4.7 g/L, *P* = 0.043), and the pre-TIPS creatinine level was significantly lower in the combination group than in the Viatorr group (65.3 ± 23.7 μmol/L vs. 80.6 ± 40.7 μmol/L, *P* = 0.005). There were no significant differences in other baseline characteristics between the two groups.

**Table 1:** Baseline of patients.

Variables	All patients (N = 145)	Combination group (N = 82)	Viatorr group (N = 63)	*P* values
Demographic characteristics				
Age, years	58.1 ± 11.3	57.3 ± 11.4	59.3 ± 11.1	0.290
Gender, male	83 (57.2)	42 (51.2)	41 (65.1)	0.094
Body weight, kg	61.1 ± 10.8	60.5 ± 11.3	62.0 ± 10.2	0.412
Height, m	1.6 ± 0.1	1.6 ± 0.1	1.6 ± 0.1	0.079
BMI, kg/m^2^	22.6 ± 4.1	22.9 ± 4.2	22.4 ± 4.1	0.482
Indications for TIPS				0.677
Variceal bleeding	124 (85.5)	71 (86.6)	53 (84.1)	
Refractory ascites	21 (14.5)	11 (13.4)	10 (15.9)	
Etiology				0.561
HBV	96 (66.2)	56 (68.3)	40 (63.5)	
HCV	16 (11.0)	10 (12.2)	6 (9.5)	
Alcohol	7 (4.8)	3 (3.7)	4 (6.3)	
*Schistosoma*	7 (4.8)	5 (6.1)	2 (3.2)	
Others	19 (13.0)	8 (9.8)	11 (17.5)	
Laboratory parameters				
TBIL, μmol/L	28.4 ± 29.7	24.8 ± 18.2	33.2 ± 39.8	0.093
ALB, g/L	30.3 ± 5.2	29.5 ± 5.4	31.3 ± 4.7	0.043
ALT, U/L	36.4 ± 71.5	28.9 ± 22.3	46.2 ± 105.2	0.152
AST, U/L	51.2 ± 76.7	46.3 ± 47.0	57.5 ± 95.0	0.356
Creatinine, μmol/L	71.9 ± 32.9	65.3 ± 23.7	80.6 ± 40.7	0.005
BUN, mmol/L	6.9 ± 4.2	6.8 ± 4.0	7.1 ± 4.4	0.652
PT, seconds	17.0 ± 3.2	16.7 ± 2.5	17.4 ± 4.0	0.196
INR	1.5 ± 0.4	1.4 ± 0.3	1.5 ± 0.4	0.157
Platelet count, 10^9^/L	92.5 ± 84.5	96.8 ± 83.2	86.7 ± 86.6	0.483
Sodium, mmol/L	138.8 ± 4.5	138.5 ± 3.7	139.2 ± 5.3	0.340
NH_4_^+^	45.8 ± 33.2	45.7 ± 35.3	46.0 ± 30.9	0.962
Radiographic analysis				
SPSS, yes	26 (17.9)	15 (18.3)	11 (17.5)	0.897
PVT, yes	52 (35.9)	28 (34.1)	24 (38.1)	0.623
Portal vein diameter, mm	15.3 ± 3.8	14.9 ± 3.6	15.9 ± 4.0	0.163
Splenic vein diameter, mm	10.6 ± 3.2	10.4 ± 3.6	10.8 ± 2.6	0.631
Pre-TIPS PPG, mmHg	26.2 ± 5.2	26.3 ± 5.7	26.1 ± 5.1	0.925
Scores				
CTP	6.9 ± 2.0	7.0 ± 1.9	6.7 ± 2.0	0.474
MELD	12.1 ± 4.7	11.6 ± 3.8	12.7 ± 5.7	0.156
MELD-Na	12.4 ± 6.0	12.2 ± 5.1	12.5 ± 7.1	0.768
FIPS	− 1.1 ± 0.4	− 1.1 ± 0.9	− 1.0 ± 0.7	0.683

BMI, body mass index; HBV, hepatitis B virus; HCV, hepatitis C virus; TBIL, total bilirubin; ALB, albumin; ALT, alanine aminotransferase; AST, aspartate aminotransferase; BUN, blood urea nitrogen; PT, prothrombin time; INR, international normalized ratio; SPSS, spontaneous portosystemic shunts; PVT, portal vein thrombosis; TIPS, transjugular intrahepatic portosystemic shunt; PPG, porto-caval pressure gradient; CTP, Child-Turcotte-Pugh; MELD, model for end-stage liver disease; FIPS, Freiburg index of post-TIPS survival.

### Details of TIPS creation

Among the total 145 patients, 41 (28.3%) used auxiliary targeting techniques to guide puncture, with 29 (35.4%) in the combination group and 12 (19.0%) in the Viatorr group (Table 2). The proportion of patients in the Viatorr group who used a 6mm balloon for dilation was higher than that in the combination group (74.4% vs. 88.9%, *P* = 0.028). The operation duration in the Viatorr group was shorter than that in the combination group (89.5 ± 51.7 min vs. 71.3 ± 34.1 min, *P* = 0.017). Hospital length of stay was similar between the two groups, with no statistically significant difference (10.5 ± 6.8 d vs. 12.7 ± 11.8 d, *P* = 0.155). In the combination group, 47 (57.3%) patients used Fluency stents, and 35 (42.7%) patients used Viabahn stents.

**Table 2:** Details of TIPS procedure.

Variables	All patients (N = 145)	Combination group (N = 82)	Viatorr group (N = 63)	*P* values
Shunt dysfunction, yes	11 (7.6)	8 (9.8)	3 (4.7)	0.350
Balloon diameter, 6 mm	117 (80.7)	61 (74.4)	56 (88.9)	0.028
Varicose vein embolism	89 (61.4)	48 (58.5)	41 (65.1)	0.497
Auxiliary target, yes	41 (32.4)	29 (35.4)	12 (19.0)	0.031
Operation duration, min	81.6 ± 45.6	89.5 ± 51.7	71.3 ± 34.1	0.017
Hospital stays, d	11.5 ± 9.4	10.5 ± 6.8	12.7 ± 11.8	0.155
Min-stent diameter, mm	6.3 ± 1.4	6.2 ± 1.7	6.5 ± 1.1	0.289
Post-TIPS PPG, mmHg	11.8 ± 5.6	11.7 ± 5.8	11.9 ± 5.6	0.833
ΔPPG, mmHg	14.5 ± 5.0	15.0 ± 5.4	14.2 ± 4.9	0.599
α angle	39.7 ± 16.9	41.2 ± 18.2	36.9 ± 14.9	0.094
β angle	107.4 ± 65.0	102.9 ± 64.2	113.1 ± 66.2	0.398

The α and β angles refer to the angle of deviation of the blood flow at the portal venous inflow and central venous outflow, respectively. Specifically, the α angle is the angle between the stent and the portal vein, while the β angle is the angle between the stent and the hepatic vein.

**Figure 3:**
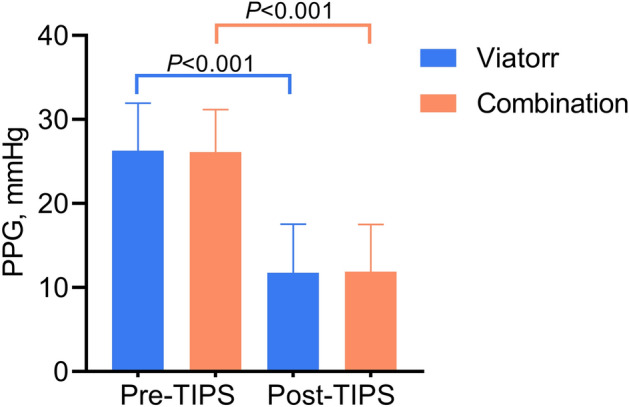
Changes of PPG in patients before and after TIPS creation.

TIPS creation was technically successful in all cases. After TIPS creation, the PPG in both the combination and Viatorr groups decreased significantly (Fig. 3). In this study, 57% of patients had post-TIPS PPG below 12 mmHg, and 43% had post-TIPS PPG above 12 mmHg, but decreased by more than 20%. Specifically, in the combination group, the PPG significantly decreased compared to pre-TIPS levels (26.3 ± 5.7 mmHg vs. 11.7 ± 5.8 mmHg, *P* < 0.001). Similarly, in the Viatorr group, the PPG significantly decreased compared to pre-TIPS levels (26.1 ± 5.1 mmHg vs. 11.9 ± 5.6 mmHg, *P* < 0.001). There were no significant differences in the α and β angles between the two groups of patients after TIPS placement. No complications occurred in any patient post-TIPS. Among the patients in the entire study cohort, 11 (7.6%) experienced shunt dysfunction, including 8 (9.8%) in the combination group and 3 (4.7%) in the Viatorr group. Out of the 11 patients, only 3 (1 in the combination group and 2 in the Viatorr group) required stent revision (new stent placement), while the remaining 8 underwent balloon dilatation plasty.

### Clinical outcomes

**Figure 4:**
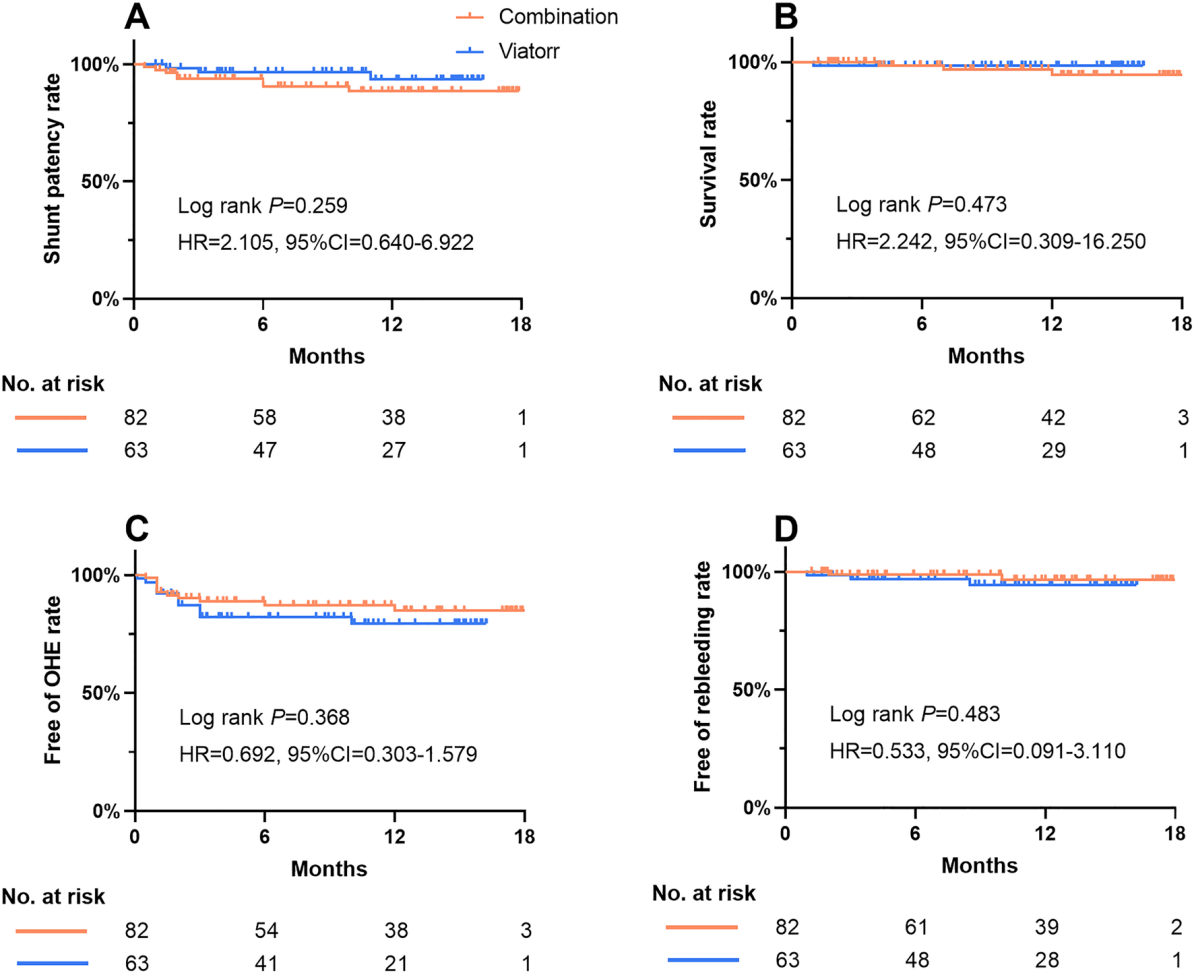
KM curves for different study endpoints. (**A**) shunt dysfunction. (**B**), death. (**C**), OHE. (**D**), rebleeding.

Outcomes of the two groups of patients were compared and analyzed using KM curves, including shunt dysfunction, death, rebleeding, and OHE (Fig. 4). With shunt dysfunction as the study endpoint, the median follow-up time for the combination and Viatorr groups was 10.8 months (IQR 4.7–14.0) and 10.6 months (IQR 4.6–14.9), respectively. The shunt patency rates were reported at 6, 12, and 18 months for both the combination and Viatorr groups. The shunt patency rates for the combination and Viatorr groups were 93.7% and 96.7%, respectively, at 6 months, 88.5% and 93.4%, respectively, at 12 months, and 88.5% and 93.4%, respectively, at 18 months. The KM curve showed no significant difference in the shunt patency rates between the combination and Viatorr groups (HR = 2.105, 95% CI 0.640–6.922, Log-rank *P* = 0.259). There were no significant differences in other outcomes between the two groups, including survival rate (HR = 2.242, 95% CI 0.309–16.250, Log-rank *P* = 0.473), rebleeding rate (HR = 0.533, 95% CI 0.091–3.110, Log-rank *P* = 0.483), and OHE rate (HR = 0.368, 95% CI 0.303–1.579, Log-rank *P* = 0.368).

### Independent risk factors for shunt dysfunction

**Figure 5:**
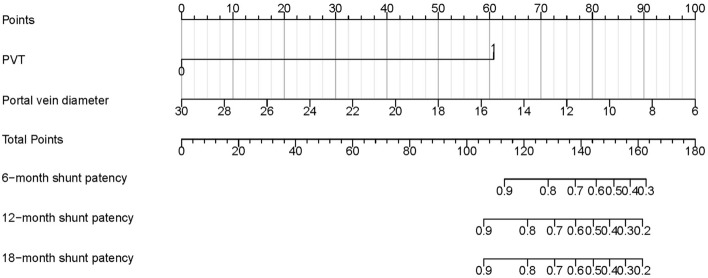
A nomogram for predicting shunt dysfunction after TIPS creation.

Predictors for shunt dysfunction were determined using the Cox proportional hazards model (Table 3). The variables with a *P*-value < 0.2 in the univariate analysis were included in the multivariate analysis after adjusting for confounding factors. The variables included in the multivariate analysis were gender, aspartate aminotransferase, international normalized ratio, portal vein diameter, PVT, and Viatorr stent. The multivariate Cox analysis identified portal vein diameter (HR = 0.807, 95% CI 0.658–0.990, *P* = 0.040) and PVT (HR = 13.617, 95% CI 1.475–125.678, *P* = 0.021) as predictors for shunt dysfunction. Viatorr stent (HR = 0.310, 95% CI 0.061–1.575, *P* = 0.158) was not identified as a predictor for shunt dysfunction. Based on the results of the multivariate analysis, a nomogram was created to predict the likelihood of shunt dysfunction after TIPS creation (Fig. 5). Using the nomogram, clinicians can predict the risk of shunt dysfunction post-TIPS in patients. For patients at high risk, clinicians should pay closer attention to their prognosis.

**Table 3:** Independent risk factors for shunt dysfunction.

Variables	Univariate			Multivariate		
	*P*	HR	95%CI	*P*	HR	95%CI
Age, years	0.345	0.975	0.925–1.027			
Gender, male	0.110	3.499	0.754–16.229	0.092	6.149	0.742–50.931
BMI, kg/m^2^	0.726	1.028	0.881–1.199			
TBIL, μmol/L	0.465	0.985	0.947–1.025			
ALB, g/L	0.700	0.978	0.872–1.096			
ALT, U/L	0.414	0.983	0.943–1.025			
AST, U/L	0.196	0.972	0.973–1.015	0.806	0.997	0.970–1.024
Creatinine, μmol/L	0.358	0.986	0.957–1.016			
BUN, mmol/L	0.251	0.884	0.716–1.091			
PT, seconds	0.143	0.780	0.560–1.087			
INR	0.102	0.083	0.004–1.636	0.744	0.585	0.023–14.639
Platelet count, 10^9^/L	0.473	1.002	0.997–1.007			
Sodium, mmol/L	0.536	0.960	0.845–1.092			
SPSS, yes	0.388	0.402	0.051–3.177			
Portal vein diameter, mm	0.167	0.878	0.731–1.056	0.040	0.807	0.658–0.990
PVT, yes	0.006	8.622	1.862–39.926	0.021	13.617	1.475–125.678
Pre-TIPS PPG, mmHg	0.433	1.065	0.910–1.247			
Post-TIPS PPG, mmHg	0.604	1.044	0.888–1.226			
ΔPPG, mmHg	0.861	1.015	0.858–1.201			
Viatorr stent, yes	0.271	0.475	0.126–1.790	0.158	0.310	0.061–1.575
α angle	0.635	1.011	0.967–1.057			
β angle	0.811	1.001	0.990–1.012			

BMI, body mass index; TBIL, total bilirubin; ALB, albumin; ALT, alanine aminotransferase; AST, aspartate aminotransferase; BUN, blood urea nitrogen; PT, prothrombin time; INR, international normalized ratio; SPSS, spontaneous portosystemic shunts; PVT, portal vein thrombosis; TIPS, transjugular intrahepatic portosystemic shunt; PPG, porto-caval pressure gradient; HR, hazard ratio; CI, confidence interval.

## Discussion

Transjugular intrahepatic portosystemic shunt is an effective method of treating portal hypertension by creating a direct connection between the hepatic vein and the portal vein^12^. In recent times, the development of stents has advanced and promoted TIPS creation. The Viatorr stent has been widely used globally^13^. However, due to its high cost, it is not widely used in some regions with high disease burden, such as China, Japan, and so on^14^. A combination of covered and bare stents can also result in a better patient prognosis^7^. We analyzed TIPS cases in our center in the past 2 years, aiming to compare the Viatorr stent and combination stents to determine which provided greater survival benefits for patients. Importantly, our research was not about comparing stents from different manufacturers but rather comparing specialized stents to combination stents. We found that both the combination group and the Viatorr group had high shunt patency rates at 6, 12, and 18 months (93.7% vs. 96.7%, 88.5% vs. 93.4%, 88.5% vs. 93.4%, respectively). The shunt patency rates in the Viatorr group were higher than that in the combination group but not statistically significant (HR = 2.105, 95% CI 0.640–6.922, Log-rank *P* = 0.259). In addition, there were no significant differences between the two groups in terms of survival, OHE, and rebleeding. Furthermore, we found that using the Viatorr stent resulted in less operation duration (71.3 ± 34.1 min vs. 89.5 ± 51.7 min, *P* = 0.017).

The Viatorr stent was a specialized stent widely used for constructing the shunt between the portal vein and hepatic vein in TIPS. When the availability of Viatorr stents was limited, combination stents can be used as an alternative^7^. Our study confirmed that the combination stent method can achieve good therapeutic effects. During the 18 months, the cumulative rate of survival, shunt dysfunction, OHE, and rebleeding was not significantly different from those of the Viatorr group. Previous studies have also indicated that Viatorr and Fluency have similar cumulative patency rates (89% and 81% at 1 year, respectively, *P* = 0.03)^15^. In our study, the Viatorr and combination groups had 1-year cumulative patency rates of 93.4% and 88.5%, respectively, both higher than those reported in previous studies^16,17^. The Viatorr group had a slightly higher cumulative patency rate than the combination group in our study, but there was no statistically significant difference, which may be due to differences in race and sample size. Due to the prevalence of hepatitis B in China, TIPS creation was mainly indicated for gastrointestinal bleeding. In contrast, another study comparing Viatorr with Fluency showed that the Viatorr group had a significantly higher 5-years primary patency rate than the Fluency group (89.0% vs. 19.6%, *P* < 0.001)^18^. The difference may be since Fluency was mainly used without the routine combination with bare stents, while the use of bare stents can maintain intrahepatic portal vein perfusion according to the portal vein’s course. There was a lack of studies on clinical efficacy comparing Viabahn with bare stents and Viatorr.

The Viatorr stent consists of a 4–8 cm covered segment and a 2 cm bare segment of the portal vein^14^. Unlike the Fluency stent, the expanded polytetrafluoroethylene aperture size of the covered segment of Viatorr is smaller, making it less susceptible to bile leakage during and after TIPS creation^19^. Bile leakage was associated with pseudo intimal hyperplasia and thrombus formation^20^. Fluency often lacks the flexibility needed to bend its distal end, which can lead to poor adherence to blood vessels^21,22^. During placement, a “cap” may form if the stent was located too low at the end of the hepatic vein or too short at the end of the portal vein, leading to poor blood flow and stent dysfunction^23^. Viabahn, which was also used as the covered part of combination stents, had good flexibility and bend resistance, and resist rebounding after placement^24^. However, the radial supporting force of Viabahn was weak, which may result in shortening. Whether a dedicated stent or combination stents was chosen, perfect coverage over the intrahepatic shunt channel would greatly reduce post-TIPS thrombus formation. Locating the boundary between the intrahepatic shunt channel and the portal vein puncture site was often difficult under digital subtraction angiography, and precisely measuring the shunt channel length to choose the most appropriate stent was also a challenge. When placing a combination stent, the bare stent and the covered stent were placed separately, making it often difficult to precisely cover the covered stent over the intrahepatic shunt channel. Viatorr had a golden ring marking for accurate positioning of the covered and bare segments, which required high experience from interventionists^25^.

In our study, all three cases of shunt dysfunction in the Viatorr group were located at the hepatic vein end, which was consistent with previous research findings^15^. The proportion of Viatorr stent placement with stenosis at the hepatic vein end ranged from 43 to 100%^26–29^. Some studies suggest that extending the Viatorr stent to the junction between the hepatic vein and inferior vena cava can reduce hepatic vein end stenosis^15^. Ideally, the stent selected based on the length of the shunt should extend to the junction between the hepatic vein and inferior vena cava. However, in practice, some patients required additional stents to achieve the necessary length. In our study, one patient in the Viatorr group required an additional stent. Although this undoubtedly increased the cost, it was gratifying that this patient did not experience stent malfunction during the follow-up period. Out of the 8 patients with stent dysfunction in the combination group, one case was blocked off at the hepatic vein end, while the other 7 cases were blocked off at the portal vein end. This finding highlighted the difference between the two stent techniques.

Our study found that portal vein diameter and PVT were independent risk factors for stent malfunction, which was consistent with previous studies^30–32^. A smaller portal vein diameter and complete or extensive PVT before TIPS increased the risk of stent malfunction by decreasing blood flow into the TIPS shunt channel, potentially leading to stent occlusion^33^. In a TIPS study of 51 patients with PVT, the degree of PVT and superior mesenteric vein thrombosis were associated with TIPS failure and shunt dysfunction^30^. Therefore, paying extra attention to the condition of the portal vein in patients may help reduce the occurrence of post-TIPS stent malfunction.

However, this study has some limitations. Firstly, as a retrospective study, there was inevitably a selection bias. In addition, the majority of patients who received TIPS creation due to varicose vein bleeding accounted for, while in Europe and the United States, this proportion was not so high. Besides, the relatively short follow-up time of 2 years maximum did not reflect differences in long-term prognosis between the specialized stent-graft and generic stent-graft/bare stent combination at 3 or even 5 years, as the use of Viatorr stents in our medical center was still relatively new.

## Conclusion

The shunt patency rates between the Viatorr stent and the generic stent-graft/bare stent combination showed no significant difference and the generic stent-graft/bare stent combination may be a viable alternative in areas where the Viatorr stent is not yet available.

## Data Availability

The datasets used during the current study available from the corresponding author on reasonable request.

## Abbreviations

HBV: Hepatitis B virus
HCV: Hepatitis C virus
TBIL: Total bilirubin
ALB: Albumin
ALT: Alanine aminotransferase
AST: Aspartate aminotransferase
BUN: Blood urea nitrogen
PT: Prothrombin time
INR: International normalized ratio
SPSS: Spontaneous portosystemic shunts
PVT: Portal vein thrombosis
TIPS: Transjugular intrahepatic portosystemic shunt
PPG: Portal pressure gradient
